# Th1 and Th17 hypercytokinemia as early host response signature in severe pandemic influenza

**DOI:** 10.1186/cc8208

**Published:** 2009-12-11

**Authors:** Jesus F Bermejo-Martin, Raul Ortiz de Lejarazu, Tomas Pumarola, Jordi Rello, Raquel Almansa, Paula Ramírez, Ignacio Martin-Loeches, David Varillas, Maria C Gallegos, Carlos Serón, Dariela Micheloud, Jose Manuel Gomez, Alberto Tenorio-Abreu, María J Ramos, M Lourdes Molina, Samantha Huidobro, Elia Sanchez, Mónica Gordón, Victoria Fernández, Alberto del Castillo, Ma Ángeles Marcos, Beatriz Villanueva, Carlos Javier López, Mario Rodríguez-Domínguez, Juan-Carlos Galan, Rafael Cantón, Aurora Lietor, Silvia Rojo, Jose M Eiros, Carmen Hinojosa, Isabel Gonzalez, Nuria Torner, David Banner, Alberto Leon, Pablo Cuesta, Thomas Rowe, David J Kelvin

**Affiliations:** 1 National Centre of Influenza, Hospital Clínico Universitario de Valladolid, Avda Ramón y Cajal 3, Valladolid, 47005, Spain; 2 Unidad de Investigación en Infección e Inmunidad- Microbiology Service,. Hospital Clínico Universitario de Valladolid- IECSCYL, Avda Ramón y Cajal 3, Valladolid, 47005, Spain; 3 Virology Laboratory, Hospital Clinic de Barcelona, Carrer de Casanova 143, Barcelona, 08036, Spain; 4 Critical Care Department, Joan XXIII University Hospital-CIBERes Enfermedades Respiratorias-IISPV. Mallafre Guasch 4, Tarragona, 43007, Spain; 5 Critical Care Department, Hospital Universitario La Fe, Avda Campanar 21, Valencia, 46009, Spain; 6 Microbiology Service, Hospital Son Llatzer, Ctra. Manacor, km 4, Palma de Mallorca 07198, Spain; 7 Intensive Care Unit, Hospital General San Jorge, Avenida Martínez De Velasco 36, Huesca, 22004ý, Spain; 8 Intensive Care Unit & Internal Medicine Service, Hospital Gregorio Marañón, C/Doctor Esquerdo 46, Madrid, 28007, Spain; 9 Microbiology Service, Hospital Universitario de Canarias, Carretera Del Rosario 145, Santa Cruz De Tenerifeý, 38009, Spain; 10 Microbiology Service, Hospital General de La Palma, Buenavista de Arriba, s/n, Breña Alta, 38713, Spain; 11 Intensive Care Unit, Hospital Universitario de Canarias, Carretera Del Rosario 145, Santa Cruz De Tenerifeý, 38009, Spain; 12 Intensive Care Unit, Hospital Virgen del Rocío, Avenida Manuel Siurot s/n, Sevilla, 41013, Spain; 13 Intensive Care Unit Service, Hospital Son Llatzer, Ctra. Manacor, km 4, Palma de Mallorca, 07198, Spain; 14 Intensive Care Unit Service, Hospital Lozano Blesa, Avenida San Juan Bosco 15, Zaragozaý,50009, Spain; 15 Microbiology Service, Hospital Universitario Ramón y Cajal & CIBERESP, Carretera Colmenar Viejo KM 9,100, Madrid, 28049, Spain; 16 Intensive Care Unit, Hospital Universitario Ramón y Cajal, Carretera Colmenar Viejo KM 9,100, Madrid, 28049, Spain; 17 Infectious Diseases Service, Hospital Clínico Universitario, Avda Ramón y Cajal 3, Valladolid, 47005, Spain; 18 Preventive Medicine Service, Hospital Universitario Valle Hebron & CIBERESP, Paseo Vall d'Hebron, 119-129, Barcelona, 08035, Spain; 19 Experimental Theraputics Division, University Health Network, Medical Discovery Tower, 3rd floor Room 913-916,101 Collegue Street, Toronto, ON M5G 1L7, Canada; 20 International Institute of Infection and Immunity, Shantou University, 22 Xinling Road, Shantou, Guangdong Province, 515031, PR China; 21 Intensive Care Unit, Hospital de Villarobredo, Avenida Miguel de Cervantes s/n, Villarrobledo, 02600, Spain; 22 Department of Immunology, University of Toronto, Medical Discovery Tower, 3rd floor Room 913-916,101 Collegue Street, Toronto, ON M5G 1L7, Canada

## Abstract

**Introduction:**

Human host immune response following infection with the new variant of A/H1N1 pandemic influenza virus (nvH1N1) is poorly understood. We utilize here systemic cytokine and antibody levels in evaluating differences in early immune response in both mild and severe patients infected with nvH1N1.

**Methods:**

We profiled 29 cytokines and chemokines and evaluated the haemagglutination inhibition activity as quantitative and qualitative measurements of host immune responses in serum obtained during the first five days after symptoms onset, in two cohorts of nvH1N1 infected patients. Severe patients required hospitalization (n = 20), due to respiratory insufficiency (10 of them were admitted to the intensive care unit), while mild patients had exclusively flu-like symptoms (n = 15). A group of healthy donors was included as control (n = 15). Differences in levels of mediators between groups were assessed by using the non parametric U-Mann Whitney test. Association between variables was determined by calculating the Spearman correlation coefficient. Viral load was performed in serum by using real-time PCR targeting the neuraminidase gene.

**Results:**

Increased levels of innate-immunity mediators (IP-10, MCP-1, MIP-1β), and the absence of anti-nvH1N1 antibodies, characterized the early response to nvH1N1 infection in both hospitalized and mild patients. High systemic levels of type-II interferon (IFN-γ) and also of a group of mediators involved in the development of T-helper 17 (IL-8, IL-9, IL-17, IL-6) and T-helper 1 (TNF-α, IL-15, IL-12p70) responses were exclusively found in hospitalized patients. IL-15, IL-12p70, IL-6 constituted a hallmark of critical illness in our study. A significant inverse association was found between IL-6, IL-8 and PaO2 in critical patients.

**Conclusions:**

While infection with the nvH1N1 induces a typical innate response in both mild and severe patients, severe disease with respiratory involvement is characterized by early secretion of Th17 and Th1 cytokines usually associated with cell mediated immunity but also commonly linked to the pathogenesis of autoimmune/inflammatory diseases. The exact role of Th1 and Th17 mediators in the evolution of nvH1N1 mild and severe disease merits further investigation as to the detrimental or beneficial role these cytokines play in severe illness.

## Introduction

The emergence of the new pandemic variant of influenza virus (nvH1N1) has brought renewed attention to the strategies for prevention, treatment and minimization of the social and human costs of the influenza disease [1-5]. The great majority of nvH1N1 infections are mild and self-limiting in nature [6-8]. Nevertheless, a small percentage of the patients require hospitalization and specialized attention in Intensive Care Units (ICUs) [9-12]. Many severe cases occur in healthy young adults, an age group rarely seriously affected by seasonal influenza [9-14]. While pregnancy and metabolic conditions (including obesity and diabetes) have been identified as risk factors for severe nvH1N1 disease, 40 to 50% of fatal cases have no documented underlying medical condition [11,12,14]. The new virus causes more severe pathological lesions in the lungs of infected mice, ferrets and non-human primates than seasonal human H1N1 virus [15]. The role of host immune responses in clearance of nvH1N1 or the role, if any, of host immune responses in contributing to severe respiratory pathogenesis of nvH1N1 infections is not known at this time. We have previously identified specific host immune response chemokine and cytokine signatures in severe and mild SARS CoV, H5N1 and Respiratory Syncytial Virus infections. In these studies, early host immune responses are characterized by the expression of systemic levels of chemokines, such as CXCL10, indicative of innate anti viral responses [16-19]. Severe and mild SARS and RSV illness could further be defined by chemokine and cytokine signatures involved in the development of adaptive immunity. Interestingly, de Jong *et al*. have demonstrated that *hypercytokinemia *of specific chemokines and cytokines is associated with severe and often fatal cases of human H5N1 infections [20]. To determine if host immune responses play a potential role in the evolution of mild or severe nvH1N1 illness we performed an analysis of systemic chemokine and cytokine levels in serum from severe and mild nvH1N1 patients shortly following the onset of symptoms. Interestingly, we identified cytokine signatures unique to mild and severe patients.

## Materials and methods

### Patients and controls

Both hospitalized and outpatients were recruited during the first pandemic wave in the months of July and August 2009 in 10 different hospitals within the National Public Health System of Spain.

**Figure 1:**
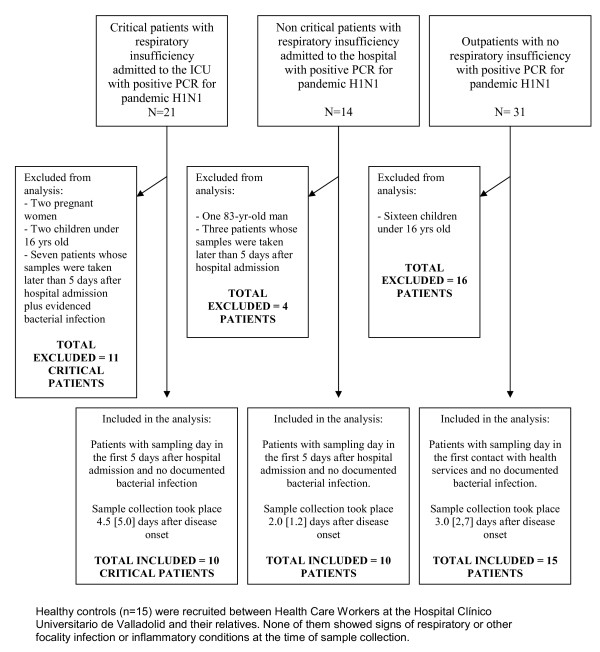
Flow chart detailing patients' recruitment and sample collection.

Inclusion criteria: Critical patients with respiratory insufficiency, hospitalized non critical patients with respiratory insufficiency, and mild outpatients with no respiratory insufficiency attending to the participant centers with confirmed nvH1N1 infection by molecular diagnostic methods (see below) were asked to donate a serum sample for the study in the first contact with the participant physicians. Initially we enrolled 35 hospitalized patients and 31 outpatients. To determine systemic levels of chemokines and cytokines in sera from nvH1N1 infected individuals, we analyzed sera from 20 hospitalized, 15 outpatients, and 15 control subjects for levels of 29 different mediators. The final number of patients used for analysis was based on exclusion and matching criteria listed in Figure 1.

Exclusion criteria: Patients with signs of bacterial infection defined by the presence of purulent respiratory secretions, and/or positive results in respiratory cultures, blood cultures, and/or positive urinary antigen test to *Legionella pneumophila *or *Streptococcus pneumoniae *were excluded from the analysis (Figure 1). Children under 16 years old and one patient older than 80 years old were also excluded in order to make groups comparable by age. Pregnant women were also excluded to avoid confusion factors during the analysis of the immune response to the virus, since pregnancy induces physiological changes in the immune system (Figure 1). Informed consent was obtained directly from each patient or their legal representative and also from the healthy controls before enrollment. Approval of the study protocol in both the scientific and the ethical aspects was obtained from the Scientific Committee for Clinical Research of the coordinating center (Hospital Clinico Universitario de Valladolid, Spain).

### Samples and laboratory studies

#### Sample collection and transport

Blood samples were collected by experienced nurses. A single serum sample was obtained from each patient or control. Serum samples were obtained after proper centrifugation and were sent refrigerated to the National Influenza Center of Valladolid (Spain), where they were stored at -70°C until immune mediator profiling, haemagglutination inhibition activity (HI) and viral load evaluation. Nasopharyngeal swabs preserved in virus transportation medium were sent to the World Health Organization (WHO) associated National Influenza Centers of Valladolid, Majadahonda and Barcelona, Spain for viral diagnosis purposes.

#### Viral diagnosis

Viral RNA from nasopharyngeal swabs was obtained by using automatic extractors (Biomerieux^® ^(Marcy l'Etoile, France), Roche^® ^(Basel, Switzerland) and viral presence was assessed by real time PCR based methods using reagents provided free of charge by the Centers for Disease Control (CDC, Atlanta, USA) or purchased from Roche^® ^(Basel, Switzerland) (H1N1 detection set) on 96-well plate termocyclers (Roche^® ^LC480 (Basel, Switzerland) and Applied Biosystems^® ^7500 (Foster City, CA, USA)

#### Viral load measurement

Viral load was measured and compared between groups by real time reverse transcription PCR on RNA extracted from serum. Briefly, an external curve was obtained by using a serial dilution of human RNA extracted from cultured monocytic leukemia (THP-1) cells, and human gene GAPDH was employed as reporter gene. nvH1N1 neuraminidase gene was amplified by QRT-PCR in each serum sample, and crossing points were extrapolated to the external curve. Analysis of samples and standard curve was conducted by using the 7500 fast v2.0.3 software (Applied Biosystem™). Results were given as relative comparisons in (pg RNA/μl). 5'-3' sequences of primer pairs: GAPDH 5'-ACCCAGAAGACTGTGGATGG-3' (forward); 5'-TTCTAGACGGCAGGTCAGGT-3' (reverse); nvH1N1 neuraminidase: 5'-TCAGTCGAAATGAATGCCCTAA-3' (forward) and N1R 5'-CACGGTCGATTCGAGCCATG-3'(reverse).

#### Cytokines and chemokines quantification

Serum chemokine and cytokine levels were evaluated using the multiplex Biorad^© ^27 plex assay (Hercules, CA, USA). This system allows for quantitative measurement of 27 different chemokines, cytokines, growth-factors and immune mediators while consuming a small amount of biological material. Furthermore, this system has good representation of analytes for inflammatory cytokines, anti-inflammatory cytokines, Th1 cytokines, Th2 cytokines, Th17 cytokines and chemokines, allowing for the testing of differential levels of regulatory cytokines in the serum of severe and mild patients. Additionally, interferon α, adiponectin and leptin were measured by using an enzyme-linked inmuno adsorbant assay (ELISA) from R&D^© ^Systems (Minneapolis, MN, USA).

#### Haemagglutination inhibition assay (HI)

HI assays were performed on a 100 μl aliquot of the samples at University Health Network (UHN), Toronto, Ontario, Canada. The sera was treated with Receptor-Destroying Enzyme (RDE) of *V. cholerae *by diluting one part serum with three parts enzyme and were incubated overnight in a 37°C water bath. The enzyme was inactivated by a 30-minute incubation at 56°C followed by the addition of six parts 0.85% physiological saline for a final dilution of 1/10. HI assays were performed in V-bottom 96-well microtiter plates (Corning Costar Co., Cambridge, MA, USA) with 0.5% turkey erythrocytes, as previously described [21], using inactivated pandemic influenza A/California/07/2009 (nvH1N1) antigens.

### Statistical analysis

Data analysis was performed using SPSS 15.0. Comparisons between groups were performed using the non parametric U-Mann Whitney test. Data are displayed as (mean, standard deviation) for clinical and laboratory parameters and as (median, interquartile rank) for data on sample collection timing and the immune mediators levels. Association between variables was determined by calculating the Spearman correlation coefficient (r) and data shown as (r, *P *value). Significance was fixed at *P *value < 0.05

## Results

### Patient's characteristics

All the patients showed symptoms of acute respiratory viral infection at disease onset. The most frequent initial symptoms were (% of patients in each group: critical, hospitalized non critical, outpatients): fever (100, 100, 80), cough (100, 90, 80), headache (90, 80, 40), tiredness (100, 80, 66) and myalgia (50, 80, 46). Hospitalized patients showed dyspnoea as the initial symptom in 90% of the cases and 100% developed respiratory insufficiency at the time of hospital admission (dyspnoea and/or hypoxemia defined as O2 saturation < 95% breathing at least two liters of oxygen). Ten patients required admission to an intensive care unit (ICU) due to their respiratory situation. The remaining 10 were admitted to other different specialized hospital services. Outpatients had no difficulties with respiratory function, showing respiratory rates under 25×'. Sex composition was the same for both critical and non critical hospitalized patients: 60% of the patients were male (n = 12) and 40% female (n = 8). Fifty-three percent of the outpatients were male and 47% female (n = 8 and 7 respectively) (Table 1). Average age was as follows: hospitalized patients (36.6; 11.5), outpatients (29.7; 8.0) and healthy controls, (29.5; 13.2). Critical patients were slightly older than the other hospitalized patients (Table 1). Seven patients with critical illness and four severe patients with non critical illness showed previous pathologies (Table 1). Ten out of 10 of the critical patients, and 6/10 of the severe non critical patients showed a pathological chest x-ray within 24 hours of onset of the symptoms (Table 1). Outpatients had received just antipyretics (paracetamol) before sample collection (none of them had received oseltamivir). One hundred percent of the hospitalized patients (critical and non critical), had received oseltamivir at the time of sample collection (Table 1). Lymphopenia was a common finding in the critical patients (mean; SD) (358.5; 267.1). LDH levels were increased over normal levels in hospitalized patients, mostly in those critically ill (Table 1). Furthermore, critical patients also showed high levels of CPK, GOT, GPT and glucose in venous blood (Table 1). Critical patients stayed longer at the hospital than the other hospitalized patients (Table 1). Three critical patients ultimately died (five days after onset due to hypoxemia and septic shock; 69 days after onset by refractory hypoxemia complicated by systemic candidiasis; and the third after 75 days of supportive therapy by multiorganic failure).

**Table 1:** Clinical and laboratory characteristics of the patients

	**Hospitalized, critical illness(n = 10)**	**Hospitalized, non critical illness (n = 10)**
	
**Pathological antecedents**		
Esquizophrenia	1/10	-
COPD	1/10	-
Diabetes	2/10	-
Asthma	-	2/10
COPD+HIV	-	1/10
Chronic disease conective tissue	-	1/10
Dyslipemia	1/10	-
Cardiopathy	1/10	-
Hypertension	1/10	-
Obesity (BMI>30)	5/10	3/10
		
**Descriptives**		
Age (yrs)	41.8 (9.9)	31.3 (10.9)
Sex (M/F)	6/4	6/4
Days at hospital	29.5 (29.2)	6.5 (2.8)
Days at ICU	26.6 (30.8)	0 (0)
		
**Severity scores**		
SOFA score	5.6 (2.9)	-
APACHEII score	12.8 (4.2)	-
		
**Respiratory condition**		
Mechanical Ventilation	9/10	0/10
O2 saturation (%)	83.3 (7.3)	93.0 (5.1)
PaO2 (mmHg)	54.1 (11.6)	76.5 (24.3)
PaO2:FiO2	94.0 (89.9)	252.5 (20.6)
		
**Opacity in initial chest X-Ray**		
0/4 quadrants	0/10	4/10
1/4 quadrants	2/10	3/10
2/4 quadrants	3/10	3/10
3/4 quadrants	0/10	0/10
4/4 quadrants	5/10	0/10
		
**Biochemistry**		
LDH (IU/liter)	1634 (1226.0)	475.1 (356.7)
CPK (IU/liter)	588.5 (606.1)	108.5 (132.1)
GOT (U/liter)	126.9 (73.5)	35.6 (14.5)
GPT (U/liter)	130.7 (97.6)	35.7 (18.9)
Glucose (mg/dl)	202.7 (97.1)	113.1 (29.1)
CRP (mg/l)	85.4 (76.3)	61.1 (105.1)
		
**Treatment received at the time of sample collection**		
Oseltamivir	10/10 (75-150 mg/12 hs)	10/10 (75 mg/12 hs)
Cephalosporines	6/10	2/10
Macrolides	3/10	1/10
Quinolones	5/10	7/10
Steroids	4/10 (parenteral)	2/10 (inhaled)
Noradrenaline	5/10	0/10
Renal replacement therapy	2/10	0/10

COPD = Chronic Obstructive Pulmonary Disease; HIV = Human Immunodeficiency Virus; BMI = Body Mass Index; ICU = Intensive Care Unit; SOFA = Sepsis-related Organ Failure Assessment score; APACHE II = Acute Physiology and Chronic Health Evaluation II; PaO2 = pressure of oxygen in arterial blood; FiO2 = fraction of inspired oxygen; LDH = Lactate dehydrogenase; CPK = creatine phosphokinase; GOT = Glutamyl oxaloacetic transaminase; GPT = Glutamyl pyruvic transaminase; CRP = C Reactive Protein; IU = International Units; U = Units.

### HI activity

HI activity (A/California/07/2009) was present in serum from only two critically ill patients of 50 and 51 years old (titres 1/1280 and 1/160 respectively) and in one 25-year-old outpatient (titre 1/160). Serum from those three patients showing HI showed also the ability to block viral replication, as assessed by microneutralization assay against A/California/07/2009 (data not shown). This data supports the notion that at the time of sampling the vast majority of the patients had yet to produce antibodies against nvH1N1 and was in the early stages of disease.

### Immune mediators profiling

**Figure 2:**
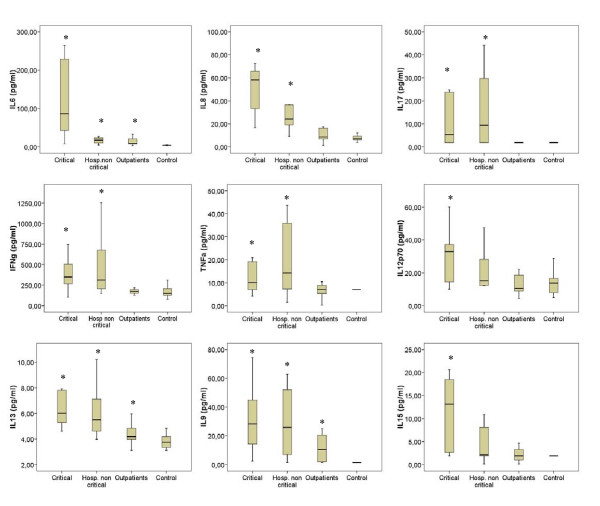
Levels of immune mediators in the four groups. *Significant differences with control at the level *P *< 0.05.

The virus induced in both mild and severe patients a systemic elevation of three chemokines that have been shown to be expressed early during viral infections, CXCL-10 (IP-10), CCL-2 (MCP-1) and CCL-4 (MIP-1β), with no differences in the levels of these mediators between them (data on immune mediators profiling are shown in Figure 2 and Additional file 1). IL-8, IFN-γ, IL-13, IL-10 levels were higher in the hospitalized patients than in outpatients and controls (*P *< 0.05). IL-9 behaved in a similar way. While both critical and non-critical hospitalized patients showed higher levels of IL-17 and TNF-α than controls, only severe non critical patients showed significant higher levels of IL-17 and TNF-α than mild. On the other hand, IL-15 and IL-12p70 increased exclusively in critical patients, who in addition showed the highest levels of IL-6 of the compared groups.

**Figure 3:**
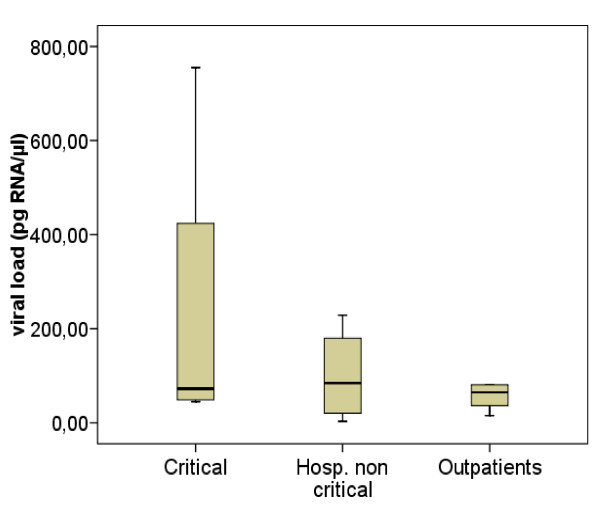
Viral load in serum. (From left to right: 0: critical patients; 1: hospitalized (non critical) patients; 2: mild outpatients). Results are expressed as (pg RNA/μl).

**Figure 4:**
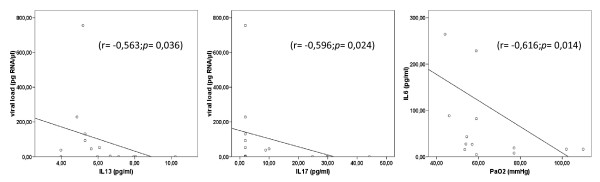
Correlation studies. From left to right: correlation between IL-13 level and viral load in serum; correlation between IL-17 level and viral load in serum; correlation between IL-6 serum levels and PaO2.

To determine if systemic viral load plays a role in chemokine or cytokine expression levels we evaluated serum for nvH1N1 levels. Fifty-seven percent of critical patients, 50% of hospitalized non critical patients, and 93% of mild patients showed positive virus in serum. For those with positive virus in serum, we found no differences in viral load between critical patients, hospitalized non critically ill, and mild outpatients (Figure 3). We found significantly higher levels of IL-13 and IL-17 in those hospitalized patients with negative virus in serum compared to those with virus in serum (data not shown). Similarly, inverse correlations were found between viral load and IL-13, IL-17 in patients requiring hospital admission (Figure 4). When mediator levels were correlated with the clinical parameters, a significant inverse association was found between IL-6 and PaO2 in hospitalized patients (Figure 4). Exclusively in the critical patients group, IL-8 inversely correlated with PaO2 [-0.7; 0.028]. In the non critically ill hospitalized patients group, a negative association was observed between IL-15 and PaO2 [-0.7; 0.039].

## Discussion

In a first attempt to understand the role host immune responses play in the evolution of severe and mild nvH1N1 disease, we assessed systemic levels of chemokines and cytokines in the sera from hospitalized and outpatients. Consistent with our previous studies on early elevated expression of CXCL10, CCL2 and CCL4 in SARS CoV and RSV infected patients [16-19], we found in the present study elevated expression of these chemokines in severe patients (critical and non critical) and mild patients. The early expression of these chemokines in all patients likely is indicative of innate antiviral host responses.

**Figure 5:**
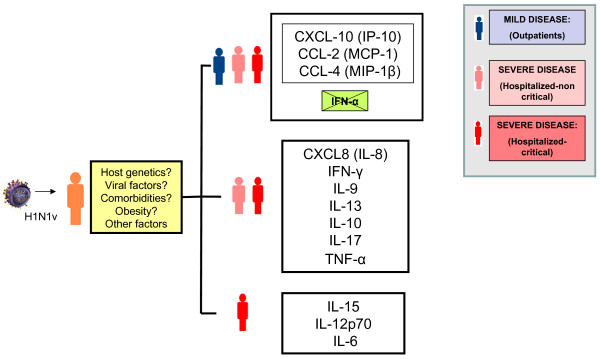
Predominant cytokine profiles paralleling early nvH1N1 disease by clinical severity.

One of the most intriguing observations in our present study is the dramatic increase of mediators which stimulate Th-1 responses (IFN-γ, TNF-α, IL-15, IL-12p70) and Th-17 ones (IL-8, IL-9, IL-17, IL-6) in the severe patients (Figure 5). Th-1 adaptive immunity is an important response against intracellular microbes such as viruses [22]. Th-17 immunity participates in clearing pathogens during host defense reactions but is involved also in tissue inflammation in several autoimmune diseases, allergic diseases, and asthma [23-27].

Increase in IFN-γ IL-8, IL-9, IL-13 and IL-10 in both critical and non critical hospitalized patients compared to mild ones indicates that they constitute hallmarks of severe disease. IFN-γ and IL-8 promote antiviral immunity but also respiratory tract inflammation by recruiting neutrophils and mononuclear cells to the site of the infection [28-30]. IL-9 is a Th2 cytokine that induces differentiation of Th-17 cells [26]. IL-10 and IL-13 show immunomodulatory properties. IL-13 attenuates Th-17 cytokine production [31]. IL-10 is known to be an anti-inflammatory cytokine. In a murine model, McKinstry *et al*.revealed that IL-10 inhibits development of Th-17 responses during influenza infection, correlating with compromised protection [32]. Increase of IL-17 and TNF-α in hospitalized patients over control indicated that they also parallel severe disease, but the significantly higher levels of IL-17 and TNF-α in severe non critical patients compared to mild (difference not found for critical ones), could reflect a beneficial role of these cytokines in this particular subset of patients. The patient who died five days after disease onset showed high viral load and undetectable IL-17 levels in serum. This could reflect a protective role of IL-17 in severe patients. IL-15, IL-12p70, IL-6 constituted a hallmark of critical illness in our study. These three cytokines also mediate both antiviral and pro-inflammatory responses. IL-6 is a potent regulator switching immune responses from the induction of Foxp3+ regulatory T cells to pathogenic Th17 cells *in vivo *[33]. IL-15 promotes CD8 T cells homeostatic proliferation [34] in response to infection. IL-12 plays a key role in the switch from innate to adaptive immunity [17].

High levels of Th-1 and Th-17 related mediators could support the hypothesis of a Th-1+Th-17 inflammatory response in the origin of the severe respiratory disease caused by nvH1N1 infection. Alternatively, an increase in Th-1 and Th-17 cytokines may reflect a vigorous antiviral host response necessary for clearance of virus during severe lower respiratory infections. While the ability of influenza A virus to induce the production of chemotactic (RANTES, MIP-1α, MCP-1, MCP-3, and IP-10) and pro-inflammatory (IL-1β, IL-6, IL-18, and TNF-α) Th1 related mediators is well know from previous reports on seasonal influenza [29,35], this is the first report evidencing Th17 response as a signature of severe influenza disease in humans [36,37]. Since there are immunomodulatory drugs which have shown to down-modulate the activity of both Th1 and Th17 [38], the results obtained here supports the development of further studies on animal models aimed to clarify the role of these mediators in the pathogenesis of the acute respiratory disease showed by severe nvH1N1 infected patients.

## Conclusions

Analysis of the immune mediators involved in host responses to the virus in mild and severe cases revealed Th1 and Th17 cytokine responses as early distinctive hallmarks of severe respiratory compromise following infection with nvH1N1. The exact role of Th1 and Th17 mediators in the evolution of nvH1N1 mild and severe disease merits further investigation as to the detrimental or beneficial role these cytokines play in severe illness. The influence of Th17-dominant conditions (autoimmune diseases) or Th1 deficient ones (HIV infection) on disease outcome should also be explored. Furthermore, the impact of other regulatory cytokines elevated in severe disease (IL-10, IL-13) on the evolution of host immune responses to nvH1N1 infections may represent alternative therapeutics for controlling severe illness.

## Key messages

• The great majority of infections caused by the new influenza pandemic virus are mild and self-limiting in nature. Nevertheless, a small percentage of the patients develop severe respiratory disease. Analysis of the immune mediators involved in host responses to the virus along with the evaluation of the humoral responses in mild and severe cases may help understand the pathogenic events leading to poor outcomes.

• Early response to the virus in both hospitalized and outpatients was characterized by expression of chemokines (CXCL10, CCL2 and CCL4), also observed in the response to SARS CoV, H5N1 and RSV, which previous literature describes to correspond to innate antiviral responses.

• Patients who develop respiratory compromise in the first days following infection with nvH1N typically showed Th1 and Th17 hyper-cytokinemia, compared to mild patients and healthy controls. These cytokine profiles have been previously reported to participate in both antiviral and pro-inflammatory responses.

• Increased systemic levels of IL-15, IL-12p70, IL-6 constituted a hallmark of critical illness. These mediators are known to promote the development of adaptive responses and also pro-inflammatory ones in other viral infections.

• Our findings constitute a major avenue to guide the design of further works studying the beneficial or detrimental role of Th1 and Th17 responses in this disease.

## Abbreviations

FGF-b: Human Fibroblast Growth Factor-basic; G-CSF: granulocyte colony-stimulating factor; GM-CSF: granulocyte macrophage colony-stimulating factor; IFN-α: interferon alpha; IFN-γ: interferon γ; IL-1RA: Interleukin 1 receptor antagonist; IP-10: Interferon-inducible protein-10; MCP-1: monocyte chemoattractant protein-1; MIP-1α: macrophage inflammatory protein-1α; MIP-1β: macrophage inflammatory protein-1β; nvH1N1; new variant of H1N1 influenza virus; PDGF-BB: platelet-derived growth factor; TNF-α: tumour necrosis factor α; VEGF: vascular endothelial growth factor.

## Competing interests

The authors declare that they have no competing interests.

## Authors' contributions

TP, JR and IML assisted in the design of the study, coordinated patient recruitment, analysed and interpreted the data, and assisted in writing the paper. PR, MCG, CS, DM, JMG, SH, ES, MG, AC, BV, CJL, JAD, CH, IG and PC supervised clinical aspects, participated in patient recruitment, assisted in the analysis, interpretation of data, and writing the report. AT, MJR, MLM, VF, MAM, MRD, JCG, RC, SR and JME performed viral diagnosis, assisted in the analysis, interpretation of data, and writing the report. RA performed cytokine profiling, and assisted in supervision of laboratory work and writing the report. NT collected clinical data, and assisted in writing the report. TR, DB performed the HAI assays and assisted in writing the report. DV and AL designed and performed the quantitative PCR method for viral load measurement. JFBM, DJK and ROL were the primary investigators, designed the study, coordinated patient recruitment, supervised laboratory works, and wrote the article.

## Supplementary Material

Additional file 1 Table listing the immune mediators' profiles in serum during the early response against the nvH1N1 virus. Click here for file
