# Supplementary material for: Th1 and Th17 hypercytokinemia as early host response signature in severe pandemic influenza
Source: Crit Care. 2009 Dec 11;13(6):R201. doi: 10.1186/cc8208 (PMC2811892; doi:10.1186/cc8208)
Supplement: Additional file 1 — Table listing the immune mediators' profiles in serum during the early response against the nvH1N1 virus. [file cc8208-S1.doc]

|  | **Critical patients (0)** | **Hosp.**  **(no crit)**  **(1)** | **Mild**  **(2)** | **Control (3)** | **0vs1** | **0vs2** | **1vs2** | **0vs3** | **1vs3** | **2vs3** |
| --- | --- | --- | --- | --- | --- | --- | --- | --- | --- | --- |
| **MCP-1** | 397.5  [954.8] | 130.0  [217.9] | 192.1  [271.6] | 38.4  [42.6] | n.s. | n.s. | n.s. | 0.000* | 0.063** | 0.000* |
| **MIP-1β** | 120.4  [174.2] | 115.1  [79.0] | 109.5  [92.6] | 61.0  [38.3] | n.s. | n.s. | n.s. | 0.002* | 0.003* | 0.003* |
| **IP-10** | 46740.6  [13706.0] | 39016.6  [193923.5] | 30154.6  [45579.9] | 1880.0  [1198.8] | n.s. | n.s. | n.s. | 0.000* | 0.000* | 0.000* |
| **FGF-β** | 8.3  [34.8] | 8.3  [74.2] | 12.9  [37.1] | 1.2  [0.0] | n.s. | n.s. | n.s. | 0.042* | 0.030* | 0.020* |
| **GM-CSF** | 13.7  [38.1] | 16.4  [32.5] | 14.5  [15.0] | 0.4  [6.1] | n.s. | n.s. | n.s. | 0.042* | 0.008* | 0.000* |
| **G-CSF** | 21.5  [28.3] | 12.0  [47.1] | 3.1  [14.4] | 1.2  [0.0] | n.s. | 0.035* | n.s. | 0.001* | 0.012* | 0.000* |
| **VEGF** | 1112.4  [1019.5] | 306.0  [638.0] | 408.1  [560.3] | 171.2  [355.5] | 0.082** | 0.014* | n.s. | 0.001* | 0.052** | 0.029* |
| **MIP-1α** | 1.5  [0.4] | 1.5  [1.2] | 1.5  [0.0] | 1.5  [0.0] | 0.088** | n.s. | 0.068** | n.s. | 0.009* | n.s. |
| **Eotaxin** | 416.8  [111.7] | 347.1  [253.9] | 414.5  [509.3] | 400.3  [145.5] | n.s. | n.s. | n.s. | n.s. | n.s. | n.s. |
| **PDGF-bb** | 46862.6  [32087.7] | 50531.3  [35907.1] | 47859.0  [44813.4] | 51420.2  [20178.6] | n.s. | n.s. | n.s. | n.s. | n.s. | n.s. |
| **IL-8** | 58.3  [38.2] | 24.2  [60.7] | 8.6  [10.2] | 7.1  [3.5] | n.s. | 0.001* | 0.006* | 0.000* | 0.000* | n.s. |
| **IL-17** | 5.3  [22.1] | 9.4  [31.5] | 1.9  [0.0] | 1.9  [0.0] | n.s. | n.s. | 0.039* | 0.029* | 0.008* | n.s. |
| **IL-6** | 86.6  [201.4] | 17.5  [13.6] | 8.9  [16.8] | 4.2  [0.9] | 0.010* | 0.002* | n.s. | 0.000* | 0.000* | 0.000* |
| **IL-9** | 28.3  [32.6] | 25.9  [47.8] | 10.5  [20.4] | 1.4  [0.0] | n.s. | 0.045* | 0.079** | 0.000* | 0.000* | 0.002* |
| **IL-13** | 6.0  [2.6] | 5.5  [2.9] | 4.2  [1.0] | 3.8  [0.9] | n.s. | 0.001* | 0.042* | 0.000* | 0.001* | 0.032* |
| **IL-4** | 3.7  [1.2] | 3.6  [4.3] | 2.5  [2.0] | 2.5  [1.6] | n.s. | 0.015* | 0.046* | 0.010* | 0.035* | n.s. |
| **IL-5** | 1.6  [0.9] | 2.3  [3.0] | 1.8  [0.6] | 1.6  [0.5] | n.s. | n.s. | 0.048* | n.s. | n.s. | n.s. |
| **IL-1β** | 1.3  [0.6] | 1.5  [1.5] | 1.0  [0.6] | 1.0  [0.2] | n.s. | n.s. | 0.055** | 0.012* | 0.001* | n.s. |
| **IFN-γ** | 349.0  [317.4] | 313.3  [470.5] | 172.3  [46.4] | 149.2  [92.8] | n.s. | 0.006* | 0.004* | 0.003* | 0.002* | n.s. |
| **TNF-α** | 10.1  [12.3] | 14.3  [30.6] | 7.1  [3.9] | 7.1  [0.0] | n.s. | n.s. | 0.037* | 0.003* | 0.001* | n.s. |
| **IL-12p70** | 33.0  [23.8] | 15.2  [18.4] | 10.5  [11.6] | 13.7  [11.0] | n.s. | 0.012* | 0.059** | 0.017* | n.s. | n.s. |
| **IL-15** | 13.1  [16.5] | 2.1  [6.9] | 1.9  [4.5] | 1.9  [0.0] | 0.048* | 0.009* | n.s. | 0.000* | n.s. | n.s. |
| **IL-2** | 1.3  [0.0] | 1.3  [1.0] | 1.3  [0.0] | 1.3  [0.0] | n.s. | n.s. | n.s. | n.s. | n.s. | 0.073** |
| **IL-10** | 22.1  [39.1] | 6.3  [2.3] | 3.8  [1.5] | 1.7  [0.6] | n.s. | 0.017* | 0.037* | 0.000* | 0.001* | 0.000* |
| **IL-7** | 20.1  [19.1] | 18.8  [13.1] | 10.1  [7.2] | 7.5  [4.1] | n.s. | 0.016* | 0.090** | 0.000* | 0.001* | 0.089 |
| **IL-1RA** | 752.2  [2580.1 | 406.3  [759.0] | 304.2  [582.4 | 29.0  [31.7] | n.s. | 0.046* | n.s. | 0.000* | 0.000* | 0.001 |
| **Adiponec** | 6211.7  [6892.8] | 4731.8  [15498.8] | 5460.8  [5453.0] | 10170.2  [13997.0] | 0.821 | n.s. | n.s. | n.s. | n.s. | n.s. |
| **Leptin** | 14740.7  [19855.2] | 1232.5  [8024.2] | 7873.5  [5822.2] | 9515.7  [5822.2] | 0.082** | 0.049* | n.s. | n.s. | n.s. | n.s. |

Results are expressed as median [interquartile range].*significant differences at the level p < 0.05; **significant differences at the level p < 0.1. (n.s).: no significant differences.
